# Phytoalexins: Current Progress and Future Prospects

**DOI:** 10.3390/molecules20022770

**Published:** 2015-02-05

**Authors:** Philippe Jeandet

**Affiliations:** Laboratory of Stress, Defenses and Plant Reproduction, Research Unit “Vines and Wines of Champagne”, UPRES EA 4707, Department of Biology and Biochemistry, Faculty of Sciences, University of Reims, P.O. Box 1039, 51687 Reims cedex 02, France; E-Mail: philippe.jeandet@univ-reims.fr; Tel.:+33-3-2691-3341; Fax: +33-3-2691-3340

Phytoalexins are low molecular weight antimicrobial compounds that are produced by plants as a response to biotic and abiotic stresses. As such they take part in an intricate defense system which enables plants to control invading microorganisms. In the 1950s, research on phytoalexins started with progress in their biochemistry and bio-organic chemistry, resulting in the determination of their structure, their biological activity, as well as mechanisms of their synthesis and catabolism by microorganisms. Elucidation of the biosynthesis of numerous phytoalexins also permitted the use of molecular biology tools for the exploration of the genes encoding enzymes of their synthesis pathways and their regulators. This has led to potential applications for increasing plant resistance to diseases. Phytoalexins display an enormous diversity belonging to various chemical families such as for instance, phenolics, terpenoids, furanoacetylenes, steroid glycoalkaloids, sulfur-containing compounds and indoles.

Research and review papers dealing with numerous aspects of phytoalexins including modulation of their biosynthesis, molecular engineering in plants, biological activities, structure/activity relationships and phytoalexin metabolism by micro-organisms are published in this issue.

In the first paper of this special issue on phytoalexins, Jeandet *et al.* present an overview of this diverse group of molecules, namely their chemical diversity, the main biosynthetic pathways and their regulatory mechanisms, fungal metabolism, phytoalexin gene transfer in plants and their role as antifungal and bactericidal agents as well as their involvement in human health [1].

General aspects of phytoalexins from the Leguminosae and Poaceae families are also discussed in this issue. Phytoalexins from sorghum and maize are presented in details by Poloni and Schirawski [2]. Sorghum produces two distinct phytoalexins belonging to the 3-deoxyanthocyanidin chemical group, apigeninidin and luteolinidin. Their biosynthetic pathways start from the flavanone naringenin according to a scheme slightly different from that of the anthocyanin route. In maize, phytoalexins are represented by members of the terpenoid class, including zealexins and kauralexins on the one hand and benzoxazinoids on the other hand, the biosynthesis of which are fully described. Biosynthesis aspects have been linked to both the elicitation and the up-regulation mechanisms of those phytoalexins. Various applications of sorghum and maize phytoalexins in plant disease resistance and health and biomedicine are also presented.

Within the Leguminosae family, the genus *Tephrosia*, a large pantropical genus composed of more than 350 species, is a source of numerous chemical constituents possessing various biological properties, including phytoalexin-like compounds. These compounds which are reviewed in this issue by Chen *et al.*, are mainly polyphenolics (flavones, flavonols, flavononols, flavans, isoflavones and chalcones), triterpenoids and sesquiterpenes [3]. Biosynthetic pathways of a number of these compounds are described as well as some of their biological activities as estrogenic, antitumor, antimicrobial, antiprotozoal and antifeedant agents.

Elucidating the molecular mechanisms of the modulation of phytoalexin biosynthesis finds applications in plant engineering for disease resistance. In this issue, Formela *et al.* report the effects of various sugars (sucrose, glucose and fructose) acting as endogenous signals on the mechanisms regulating the biosynthesis and accumulation of the lupine phytoalexin, genistein as well as the expression of other isoflavonoid biosynthetic genes [4]. Zernova *et al.* describe the transformation of soybean hairy roots with both the peanut resveratrol synthase 3 *AhRS3* gene and the resveratrol-*O*-methyltransferase *ROMT* gene [5]. Overexpression of these two genes resulted in the production of resveratrol and its methylated derivative pterostilbene and a lower necrosis of the transformed tissues (only 0 to 7%) in response to the soybean pathogen *Rhizoctonia solani* compared to the wild-type ones which exhibited about 84% necrosis. Biosynthesis of the 3-deoxyanthocyanidin phytoalexins from sorghum is reported in transgenic maize lines expressing the MYB transcription factor *yellow seed1* (*y1*), an orthologue of the maize gene *pericarp color1* (*p1*) in the work of Ibraheem *et al.* [6]. Expression of this transcription factor leads to the production of chemically modified 3-deoxyanthocyanidins and a resistance response of *Y1*-maize plants to leaf blight (*Colletotrichum graminicola*).

It is well known that treatment of plants with various biotic or abiotic agents, the so-called elicitors, can activate complex mechanisms in the cells by altering primary and secondary metabolisms in a coordinate fashion. Elicitors are also recognized as efficient inducers of phytoalexins. In this issue, Hadwiger and Tanaka report that EDTA, used at low concentrations, is a new elicitor of pisatin, a phytoalexin indicator of non-host resistance in pea [7]. Eliciting activity of EDTA seems to be linked to induction of cell DNA damage and defense-responsive genes.

The question of the function of phytoalexins as true antifungal agents still remains unanswered. Interestingly, a study of Sanzani *et al.* underline the effectiveness both *in vitro* and *in vivo* of some polyphenolic phytoalexins, namely the coumarin, scopoletin, on the reduction of green mold symptoms caused by *Penicillium digitatum* on oranges by 40 to 85% [8]. Based on these results, the authors conclude that treatment of plants with phytoalexins may represent an interesting alternative to synthetic fungicides. In another work by Hasegawa *et al.*, the activity of two rice phytoalexins, sakuranetin and momilactone A was tested *in vitro* and *in vivo* on the blast fungus *Magnaporthe oryzae*. Sakuranetin exhibits a higher antifungal activity than does momilactone A, respectively 40%–55% and 12%–17% reduction of mycelial growth [9].

To increase the fungitoxicity of phytoalexins, design and synthesis of more active phytoalexin derivatives is needed. Chalal *et al.* report in this issue the synthesis of a series of 13 *trans*-resveratrol analogues via Wittig or Heck reactions and assess their antimicrobial activity on two different grapevine pathogens, *Plasmopara viticola* and *Botrytis cinerea* [10]. Stilbenes displayed a spectrum of activity ranging from low to high, suggesting a relationship between the chemical structures of the synthesized stilbenes (number and position of methoxy and hydroxyphenyl groups) and their antimicrobial activity.

The ability of a fungal pathogen to weaken or neutralize the toxic effects of phytoalexins is one of the essential parameters determining the outcome of the interaction between this pathogen and its host plant. The necrotrophic fungus *Alternaria brassicicola* is known to detoxify brassinin, the indolic phytoalexin from the Brassicaceae family. A transcription factor *Bdtf1* is essential for brassinin detoxification and fungal host range. In this issue, Cho *et al.* show that beside this transcriptional factor, 10 putative genes were assumed to be involved in the detoxification of brassinin using a *Bdtf1*-deletion mutant of the necrotrophic fungus *A. brassicicola* [11].

Another limitation in our knowledge of phytoalexins is the difficulty in analyzing the events occurring between the plant and the pathogen under natural conditions. Some attempts to determine the actual concentrations and the nature of phytoalexins directly in plant tissues in response to invading microorganisms have been carried out using spectroscopic methods. Becker *et al.* in this issue describe mass spectrometry (ESI-FTIR-RMS) and imaging mass spectrometry techniques to evaluate the response of grapevine leaves to *P. viticola*, the causal agent of downy mildew [12]. Most importantly, molecular mapping of grapevine leaves by laser desorption/ionization mass spectrometry reveals a specific spatial distribution of some stilbene phytoalexins produced upon the infection process. To assess modifications of the phytoalexin metabolism *in planta*, global and untargeted approaches are also needed. Here, Marti *et al.* use a Liquid Chromatography-High Resolution Mass Spectrometry-based metabolomic approach to evaluate stilbene phytoalexin modifications as a response to an abiotic stress (UV-C radiations) in leaves of three different model plant species, *Cissus Antarctica* Vent*.* (Vitaceae), *Vitis vinifera* L. (Vitaceae) and *Cannabis sativa* L. (Cannabaceae) [13].

Interestingly, phytoalexins have found many applications in human health and disease. For example, Lozano-Mena *et al.* review in this issue the role of maslinic acid, a pentacyclic triterpene phytoalexin-like compound present in various natural sources such as herbal remedies as well as edible vegetables and fruits, as an antitumor, antidiabetic, antioxidant, cardioprotective, neuroprotective, antiparasitic and growth-stimulating agent both in experimental and animal models [14]. This offers perspectives for this compound to be used as a nutraceutical. Moreover, other phytoalexins such as brassinin and its derivative, homobrassinin, show marked antiproliferative activities *in vitro*. In this issue, Kello *et al.* indeed report that the inhibitory effects of the phytoalexin homobrassinin in human colorectal cancer cells is associated with apoptosis, G2/M phase arrest, deregulation of tubulin expression together with the loss of mitochondrial membrane potential, caspase-3 activation and intracellular reactive oxygen species production [15]. Smith *et al.* also demonstrate that the indolic phytoalexin, camalexin, exerts antitumor activity against prostate cancer cell lines by alterations of expression and activity of a lysosomal protease, cathepsin D [16]. Immunochemical analysis reveals cathepsin D relocalization from the lysosome to the cytoplasm according to camalexin treatment which is responsible for apoptosis in those cells. One of the most promising molecules in terms of biological benefits for humans, the resveratrol, is reviewed by McCalley *et al.* regarding its effects on intracellular calcium signaling mechanisms [17]. Resveratrol’s mechanisms of action are likely to be pleitropic and mediated by the interaction of this compound with key signaling proteins controlling cellular calcium homeostasis. The clinical relevance of resveratrol actions on excitable cells, transformed or cancer cells and immune cells was put in parallel with the molecular mechanisms affecting intra cellular calcium signaling proteins.

Lack of efficacy of some natural phytoalexins in reducing tumors has led to a number of investigations regarding the design and synthesis of more potent anticancer derivatives of known phytoalexins. Chalal *et al.* in this issue describe the synthesis of hydroxylated and methylated resveratrol derivatives using Wittig and Heck reactions as well as of ferrocenyl-stilbene analogs, with potent anticancer activities on human colorectal tumor SW480 cell lines [18]. However, weaker effects of the synthesized resveratrol derivatives were observed on the human hepatoblastoma HepG2 cells, showing the selectivity of those compounds for cancer treatment.

All the papers presented in this special issue thus underline the central role of phytoalexins in plant diseases as well as their involvement in human health and disease.
